# Extended depth of field in augmented reality

**DOI:** 10.1038/s41598-023-35819-9

**Published:** 2023-05-31

**Authors:** Sung Kyu Kim, Yongjoon Kwon, Ki-Hyuk Yoon

**Affiliations:** 1 Center for Artificial Intelligence, Korea Institute of Science and Technology, Seoul, 136-791 South Korea; 2 Department of Physics, Seoul Science High School, Seoul, 03066 South Korea

**Keywords:** Displays, Imaging and sensing

## Abstract

The 3D display device shows an image with depth information. Conventional 3D display devices based on binocular parallax can focus accurately only on the depth of a specific screen. Because the human eye has a narrow depth of field (DOF) under normal circumstances, 3D displays that provide a relatively wide range of virtual depth areas have limitations on the DOF where clear 3D images are seen. To resolve this problem, it is necessary to find the optical conditions to extend the DOF and analyze the phenomena related to it. For this, by using the Rayleigh criterion and the Strehl ratio, a criterion for this extension of the DOF is suggested. A practical optical structure that can effectively extend the DOF is devised using a flat panel display. This optical structure could be applied to AR, VR, and MR in the field of near-eye displays. From the results of this research, the fundamental optical conditions and standards are proposed for 3D displays that will provide 3D images with extended DOF in the future. Furthermore, it is also expected that these conditions and criteria can be applied to optical designs for the required performance in the development of 3D displays in various fields.

## Introduction

3D displays such as glasses-type or glasses-free stereo-type 3D displays usually provide binocular parallax^1,2^. In addition, to provide a motion parallax, the position information of observers can be used for a software process as feedback^3–6^. Using a multiview 3D display, motion parallax can also be provided optically^7–11^. In addition, when a person gazes at an object in a natural environment, the lines of sight from both eyes converge to the location of the object and create a fixation point in the fovea of the retina. At the same time, the eye adjusts the thickness of the eye crystalline lens so that the image of the retina becomes clear by focusing on the depth of convergence. In this way, the convergence-accommodation linkage action is naturally performed in the human eye.

In the case of the 3D image, a sense of depth may be provided from a binocular parallax image. 3D image recognition is achieved by combining binocular and monocular effects. For monocular effects, there is an effect of focus control. However, when observing a 3D image, the depth range of virtual objects that the human eye perceives as a clear image on the retina through accommodation is known to be approximately ± 0.3 diopter on average for a pupil width of 3 mm^12^. Therefore, if a 3D image with a focal depth greater than ± 0.3 diopters is provided from the 3D display, due to blurring of the image on the retina, an observer cannot see the overall clear 3D image from the provided 3D image with such a difference in depth. That is, it results in vergence-accommodation conflict (VAC)^13,14^. This VAC phenomenon can cause eye fatigue, so the depth of the 3D image to be expressed is inevitably limited, and the application area of the 3D image is also limited. Therefore, when the DOF is widened in a general 3D image, the monocular image can be said to be a 2D image that always shows a clear image regardless of the depth information of the image in the enlarged DOF area. However, if such a 3D image is combined with the binocular parallax of the gaze situation of both eyes, a clear image can always be seen when the gaze depth of both eyes is within the range of DOF. However, this is not a 3D image with the characteristics of a real image. But there is no problem in recognizing the 3D image of the gazing point because the observer can recognize a clear image even when viewing the 3D image point at any depth within the depth range of DOF.

The 3D display technology to settle this VAC problem should be able to control the departure of light from the virtual image depth, similar to holography technology^15,16^. Or implement a spatial display for providing 3D images as in a volumetric image display device^17–19^. These technologies can be applied for general glass-free 3D displays, but hologram technologies still have some limitations in the performance of spatial light modulators that display amplitude and phase for an application, and volumetric 3D displays also have the problem limiting the space for 3D displays. Therefore, they have considerable difficulty in the development of commercial 3D displays. Therefore, research and development of 3D displays that can provide focus adjustment information have been mainly attempted in the near-eye display (NED) area^20–22^. In NED, a number of studies have been conducted to expand the depth area in which focus adjustment is provided so that it can be utilized even if the viewing area of providing 3D images is limited. Additionally, there are various methods to satisfy focus adjustment in the full parallax method^23^, super-multiview (SMV) method^24^ and Light Field method^25,26^. In addition, a technology to change the depth of the virtual screen may be applied^27,28^. If the optical condition for DOF expansion is formed, in the case of a 3D display in the type of a Maxwellian view, even if only one viewpoint is provided to the monocular, a clear 3D image can always be viewed when the binocular gaze depth is within the depth range of DOF. In particular, the SMV method suggests the possibility of providing a clue to the focus control information by providing more than two parallax information points within an eye pupil. Just as depth information can be inferred from image information of both eyes by using binocular disparity, it starts with the assumption that a clue for artificial focus adjustment can be given by providing information on two or more disparities to a single eye so that depth information can be provided even in a single eye. In this case, the DOF of each viewpoint image forming SMV must be wide to provide artificial focus control information, and even if the focus is shifted to various depths, a clear image can be seen^29–31^. In this background, implementing a single parallax image with a wide DOF is an important factor in the realization of several types of 3D images, including SMV^32–35^. Therefore, this application can be used even if only one viewpoint is provided to the monocular in the case of a 3D display in the form of a Maxwellian view. In addition, when an optical system with a wide depth range of DOF is applied to an optical structure such as Maxwellian view, SMV, IP, and Light Field, hologram-like effects can be obtained as in reference papers^29,34,35^ applied to SMV. Accordingly, a 3D image similar to a hologram image, which is the ultimate 3D image, may be generated.

As a method of implementing a technique of DOF extension, there is the pin-mirror array type AR optics^36,37^. The pin-mirror-based technology has the advantage of being able to implement a compact optical system and expand the eye-box, but the use of the pin-mirror array also has the opposite effect of reducing the DOF expansion effect. In addition, HOE technology^38^ and DOF extension technology applying holographic retinal projection^39–42^ have been studied. These technologies showed the possibility of being applied as commercialized technologies when holographic display or HOE technology matures in the future.

As proposed by SMV, there is a possibility of focus adjustment when two or more parallax images are provided within the pupil diameter. Considering the background of this technology development, combining the concept of full parallax SMV technology with a method to implement ray-like light shape can achieve focusing and nonfocusing virtually and can be a way to solve the problem related to 3D image focus. Furthermore, if a wide DOF can be provided by adjusting the shape of light incident on the pupil, 3D images can be theoretically provided with all in focus. In this case, since the focus is always in focus, it is possible to generate a 3D image free from the focus problem related to focus control by using only one viewpoint information without satisfying the SMV condition. Such a 3D image without the focus problem can be utilized in various ways. However, the optical conditions for 3D displays in this technique have not been systematically studied on the limitations to the expansion of DOF according to the shape of light forming 3D images^26,29,43–46^.

In this research, the conditions for the range of DOF where the observer can adjust focus are studied under the consideration of the characteristics of geometrical and diffractive optics. Based on these theoretical backgrounds, simulations, and experimental results, geometrical and diffractive optical conditions related to the depth range of the focus-adjustable 3D image are derived. In this optical structure, the correlation between angular resolution and DOF expansion was derived to examine the limits of DOF for each angular resolution. A method is devised to present a standard for the possible DOF range by considering the diffraction effect and geometric defocus using the Rayleigh criterion, which is the criterion of resolution in in-focus status, and the Strehl ratio, which can be used for determining out-of-focus. And, based on this study, commercially applicable conditions of angular resolution and DOF expansion were found. Additionally, to implement these conditions, a realistic optical system applicable to AR-type NED displays is devised, and simulations and experimental verifications of the DOF expansion in the optical system are performed. These optical conditions can be applied to AR and VR optics, and can ultimately be applied to the design of an optical system that expands the DOF of a 3D display to display 3D images in a wide depth area. And the expanded DOF XR or 3D displays can alleviate the VAC phenomenon.

Therefore, in this paper, we propose a method to form a wide DOF 3D image by determining the realistic structure and focal depth limit of the optical system and verify that an extended focal depth can be realized through experimental reproduction and simulation results of the optical system. We first derive the conditions to extend DOF by using the correlation between the position of a light source in 3D space and the width of light incident on the pupil. Second, we devise the structure of an optical system capable of implementing the situation of this geometric condition. Third, by examining the diffraction phenomenon of the optical system occurring in this geometric DOF expansion, the amount of diffraction due to DOF expansion is calculated. Then, taking into account the correlation between geometric DOF expansion and diffractive optical DOF expansion in the devised and designed optical system, we obtain the conditions for maximizing DOF, from which we reach practical conditions for the implementable. Finally, we simulate these conditions and verify the basic conditions of realistic DOF expansion by constructing an optical system to test it.

## Results

### DOF expansion conditions in geometrical optics

**Figure 1:**
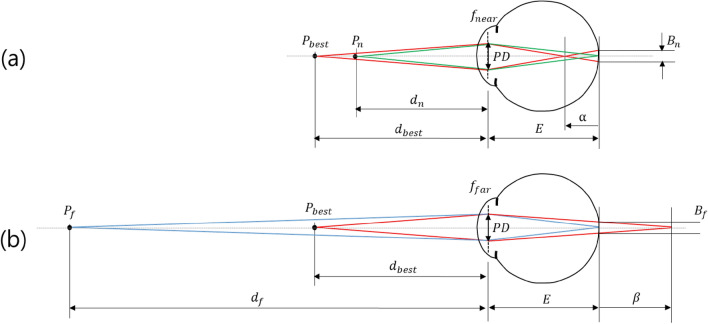
The relationship between focal depth of the eye and the optimal depth (${d}_{best}$).

If the light of each pixel constituting a 3D image can be ideally made as a light ray, it can always have a deep DOF regardless of the refractive power of the eye lens. However, this situation cannot happen in the natural environment. Light originating from any predetermined depth has a certain width as it passes through the pupil. Therefore, under the condition that light passing through the pupil forms a certain width, it is necessary to examine the DOF and its standard depth for light with the width on the pupil^45,47,48^. If the starting depth of the light source to have a focal depth for the depth area set as in Fig. 1 is ${d}_{best}$, then the depth of the virtual screen for representing the 3D image is ${d}_{best}$. Now, when an eye focuses on a 3D image point at the depth (${d}_{n}$) nearer than the virtual screen at the depth (${d}_{best}$), the image of the 3D image point at ${d}_{best}$ is formed as close as $\alpha$ before the retina, so it is blurred because the spot size of ${B}_{n}$ is formed in the retina. On the other hand, when an eye focuses on a 3D image point at a depth (${d}_{f}$) farther than the virtual screen at a depth (${d}_{best}$), the image is formed at a position farther as $\beta$ behind the retina, and it is blurred with a spot size of ${B}_{f}$ in the retina as well. Under these conditions, if the spot size related to the DOF is determined to be a certain specific value, the starting depth of the light source (${d}_{best}$) for the DOF region can be decided because both ${B}_{n}$ and ${B}_{f}$ must be equal to the determined value.

When Fig. 1 is considered, it is found that ${D}_{best}$ has a relationship of an arithmetic mean of the near distance diopter (${D}_{n}$) and the far distance diopter (${D}_{f}$) as follows:

1 $${D}_{best}=\frac{{D}_{n}+{D}_{f}}{2}.$$

The DOF range $\Delta D$ is given by

2 $$\Delta D={D}_{n}-{D}_{f}=\frac{2{B}_{f}}{PD\times \left(\frac{E}{n}\right)}=\frac{2{B}_{n}}{PD\times \left(\frac{E}{n}\right)}.$$

When light originating from the image point (${P}_{best}$) on the virtual screen passes through the eye lens, the light width on the pupil, which is the diameter of the light distribution area on the pupil, is denoted as PD.

In Eq. (2), it is shown that the DOF range is proportional to the diameter of the geometrical blur in the retina, i.e., ${B}_{n}$ or ${B}_{f}$, and inversely proportional to both the effective retinal distance, $E/n$, from the eye lens to the retina and the width of light from the object-side imaging point on the eye lens, PD. However, since the effective retinal distance is a fixed value, it can be concluded that the PD must be reduced to widen the DOF range. ${B}_{n}$ or ${B}_{f}$ can be determined by the DOF standard of the eye.

### The structure of an optical system to form an arbitrary width of light on the pupil

**Figure 2:**
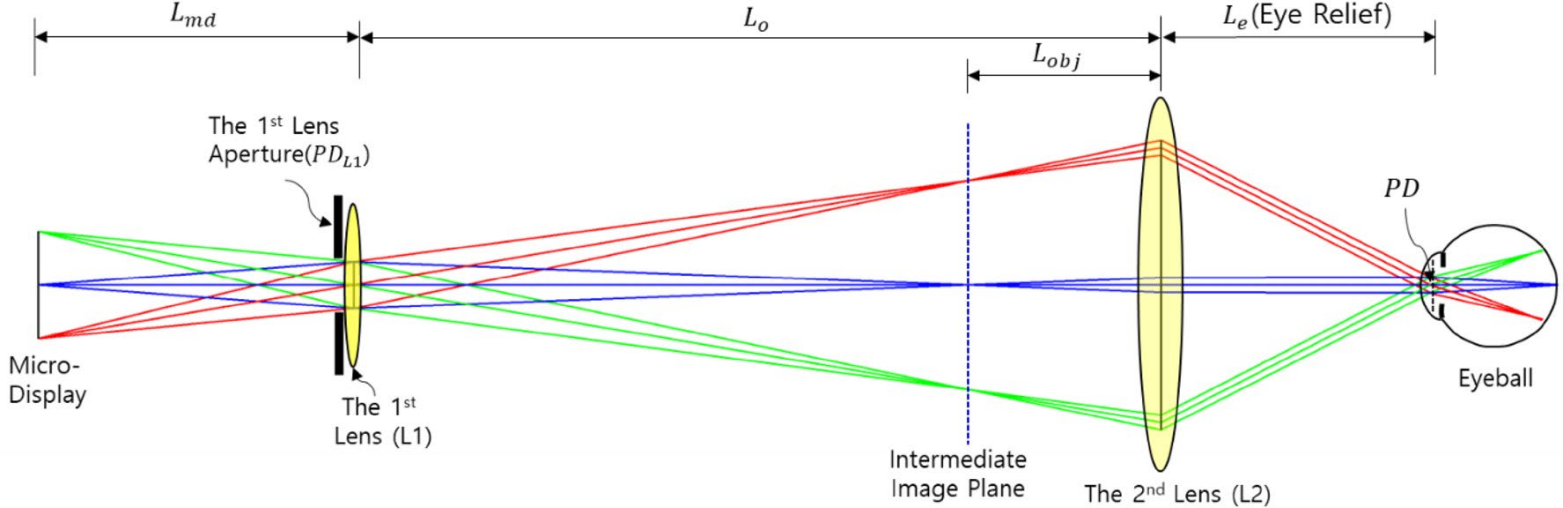
Conceptual diagram showing the basic structure of the optical system design.

Geometric optical criteria to determine the DOF region have been described in the previous section. However, there is a need for a design of an optical structure that provides a two-dimensional image capable of implementing this situation. This optical structure allows the 2D image to be viewed only when the conditions for passing through a certain area of the pupil are satisfied. In addition, since the DOF range is inversely proportional to the size of the PD on the eye lens at which the light rays generated from the imaging point arrive, a structure in which the light from the imaging point is collected on the eye lens should be constructed. Even in this convergence situation of the light, the entire virtual image of the display should be visible to the eye. Another condition is that the optical system must be configured so that the light passing through the eye lens can start at a specific optimal depth (${D}_{best}$) calculated above. The structure of the proposed optical system to satisfy these two conditions is introduced in Fig. 2. In this structure, light originating from the display passes through the 1st lens aperture associated with the light width (PD) on the eye lens and forms an image of the display within the focal distance of the second lens.

**Figure 3:**
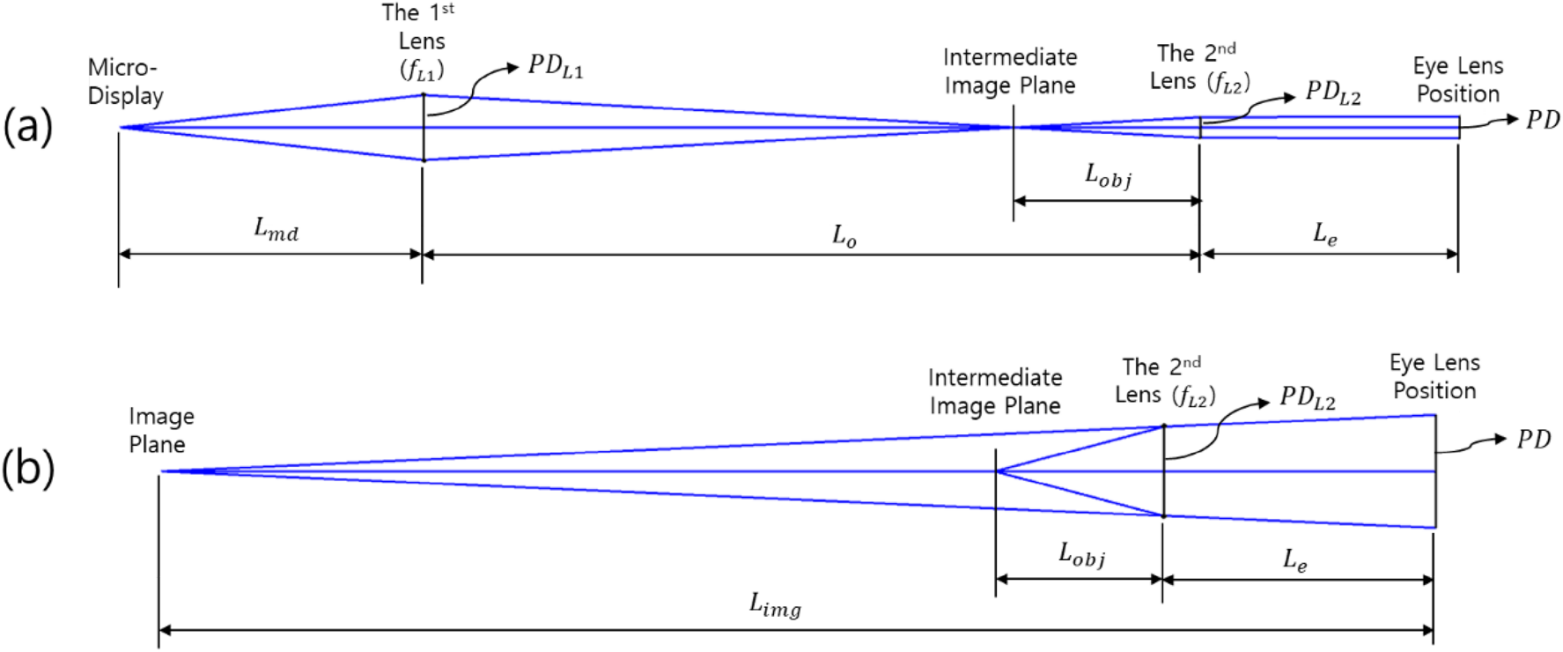
Conceptual diagram for deriving geometric relationship variables in the designed optical structure.

By setting the conditions of Figs. 2 and 3, we suggest an optical system that can determine PD and ${d}_{best}$(= ${L}_{img}$) simultaneously for the geometrically optimal DOF range. The narrower the width of light on the eye lens (PD) is, the wider the DOF range becomes. However, it should be noted that an optical system with a narrower PD inevitably increases the optical diffraction phenomenon. Therefore, the expansion of the optimal DOF range can be achieved only when these two conflicting conditions are properly compromised.

### Diffraction phenomenon at the eye for an image formed in the optical system

In this section, according to the range of geometrical DOFs, the relationship between diffraction and $PD$ is explained. When we consider the diffraction occurring in the optical system with two lenses and an eye lens designed in the previous section. When an image is formed in the retina through the optical system with three lenses, it can be shown that the size of the Airy disk is given as Eq. (3)^32,41,49^.

3 $$\text{Airy disk}=2.44\uplambda \frac{{E}_{eff}}{PD},$$

where $$\uplambda $$ represents the wavelength of light entering the eye, and $${E}_{eff}\equiv E/n$$ represents the effective retinal distance, which is the retinal distance between the eye lens and the retina divided by refractive index n. Of course, if the pupil size of the eye is smaller than $$PD$$, the pupil size can be substituted instead of $$PD$$. Therefore, the range of DOF may be defined under a condition in which the size of the geometric optical image blur ($${B}_{n}$$ or $${B}_{f})$$ in the retina, given by Eq. (2), is equal to the diffraction limit, that is, the size of the image blur due to diffraction, given by Eq. (3). Then, finally, the range of DOF is obtained as

4 $$\Delta D={D}_{n}-{D}_{f}=4.88\left(\frac{\uplambda }{{PD}^{2}}\right).$$

Note that for simplicity of the coefficient, the unit of diopter for the range of DOF ($\Delta D$), the unit of ${\text{um}}$ for the wavelength ($\uplambda$), and the unit of ${\text{mm}}$ for $PD$ are used in Eq. (4). Consequently, it is found that Eq. (4) means that the range of DOF is only inversely proportional to the square of $PD$, regardless of the structure of an optical system.

### Conceptual approach to the optical system to expand DOF

**Figure 4:**
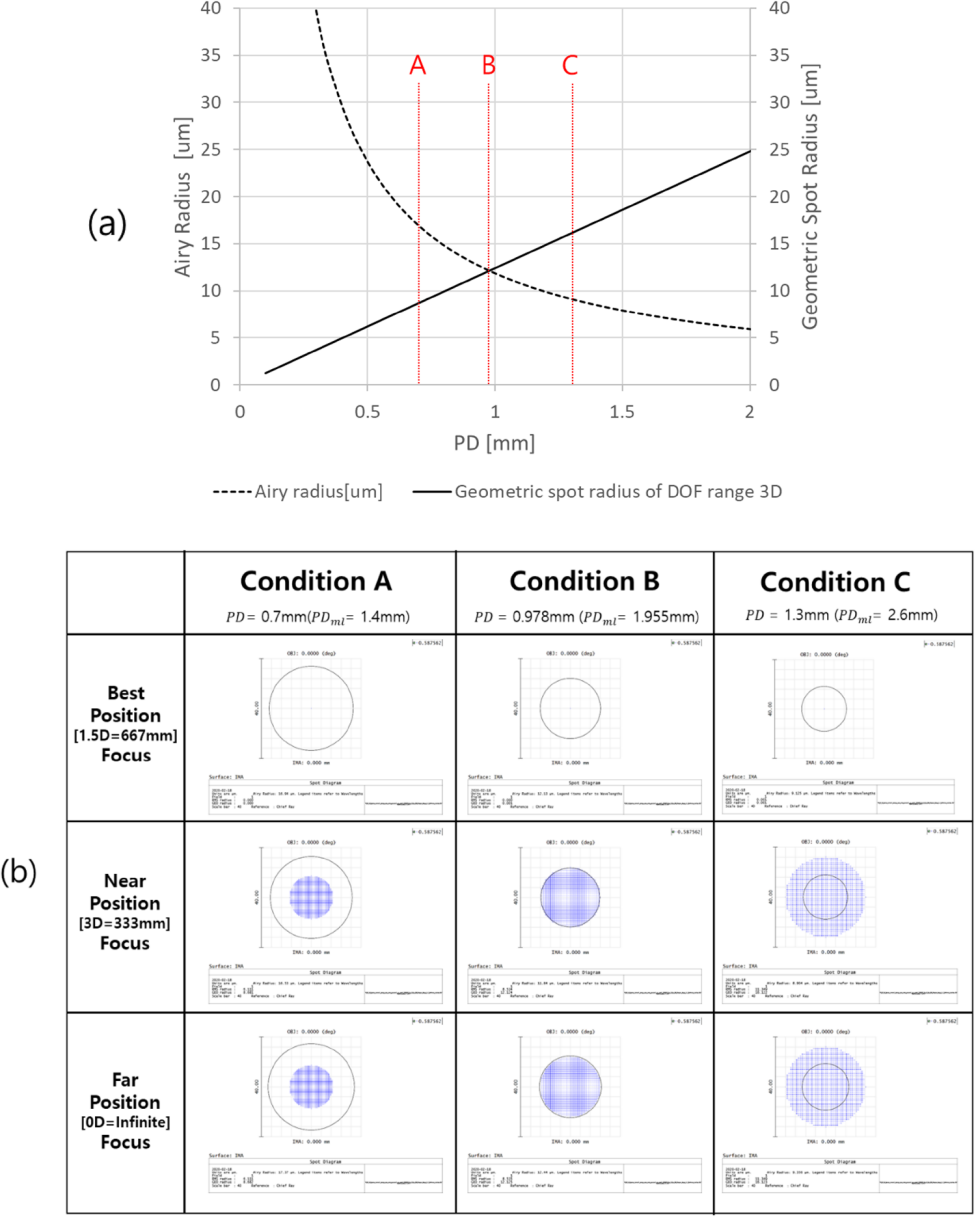
Relationship between geometrical and diffractive defocus at the aperture widths at the pupil.

To widen the DOF in AR optics as much as possible, it is explained in the previous section that the optimal trade-off between the geometrical and diffractive optical effects of the image in the retina according to PD should be found. More specifically, when PD increases, diffractive blurring decreases, as in Eq. (3) due to diffraction in the retina while the geometric blurring increases. As a result, it can be checked that a PD of approximately 1 mm corresponds to the optimum condition. For the PD of condition B in Fig. 4a, in the case that the focus of the eye lens is on the DOF boundaries of 0D and 3D, the diffraction radius and the geometric spot radius in the retina become the same for the image point of the virtual image formed in 1.5D, as shown in Fig. 4b. When the eye is focused at the DOF boundary, the PD size for condition A, which is less than the PD size for condition B, reduces the maximum resolution of the virtual image by the Airy radius compared to optimal condition B, so that it has a larger spot radius than condition B. When the eye is focused at the DOF boundary, the PD size for condition C, which is larger than the PD size for condition B, has a larger spot radius than that for condition B due to the effect of geometrical optics. As a result, the PD size on the pupil plane for which the Airy radius due to diffraction is the same as the geometric spot radius becomes an important condition to extend the DOF range.

However, this radius of Fig. 4 is based on the intensity of the geometrical and diffractive light distribution. Therefore, MFT should also be considered. From this, the resolution limit of the corresponding optical system by the diffraction limit may be determined at the reference depth (${D}_{best}$). In the following subsection, more specific conditions will be reviewed through simulation.

### Optical system design and simulation considering DOF range expansion

A method is devised to derive the PD size and implementable DOF range in consideration of the diffraction effect and geometric defocus by using the Rayleigh criterion, which is the standard for resolution in in-focus status, and the Strehl ratio, which can be used for determining out-of-focus. According to the Rayleigh criterion, the minimum of the optically perceptible interval is defined as the case where two point images are as far away as the Airy radius ($\uprho )$ in the retina, and in this case, the spatial frequency is $1/\uprho$[lp/mm]. Equation (4) gives the allowable DOF region based on the Airy radius when the focus is on, which is based on the ${d}_{best}$ depth. In this case, the image quality is degraded in both limits of $\Delta D$ compared to the ${d}_{best}$ depth, which can be checked by calculating the contrast ratio in a lens simulation program. Therefore, in terms of visual recognition, it is necessary to present a limit that does not feel a decrease in the image quality compared to the image quality at the depth of ${d}_{best}$. Although various methods may be considered for this criterion, the range of aberration due to defocusing in the eye lens may be determined as the DOF range by applying Rayleigh’s quarter-wavelength rule, which is commonly used optically^50,51^. In this case, the coefficient in Eq. (4) is changed from 4.88 to 4. When the eye focus is at the limit position of the DOF range, the Strehl ratio has a value of approximately 0.8^51–53^. Consequently, in this paper, we set a range in which the Strehl ratio by Rayleigh’s quarter-wavelength rule is 0.8 or higher as a DOF range that can be considered in-focus status without recognizing the change in the image^54,55^. The range of DOF based on the Strehl ratio is similar to the phenomenon in which the image of the lens is degraded, and as a result, the range of DOF is reduced compared to the range set based on the sizes of the geometric spot and the Airy disk. Therefore, by changing the coefficient from 4.88 to 4, Eq. (4) can be written as

5 $$PD=2\sqrt{\frac{\uplambda }{\Delta D}}.$$

**Figure 5:**
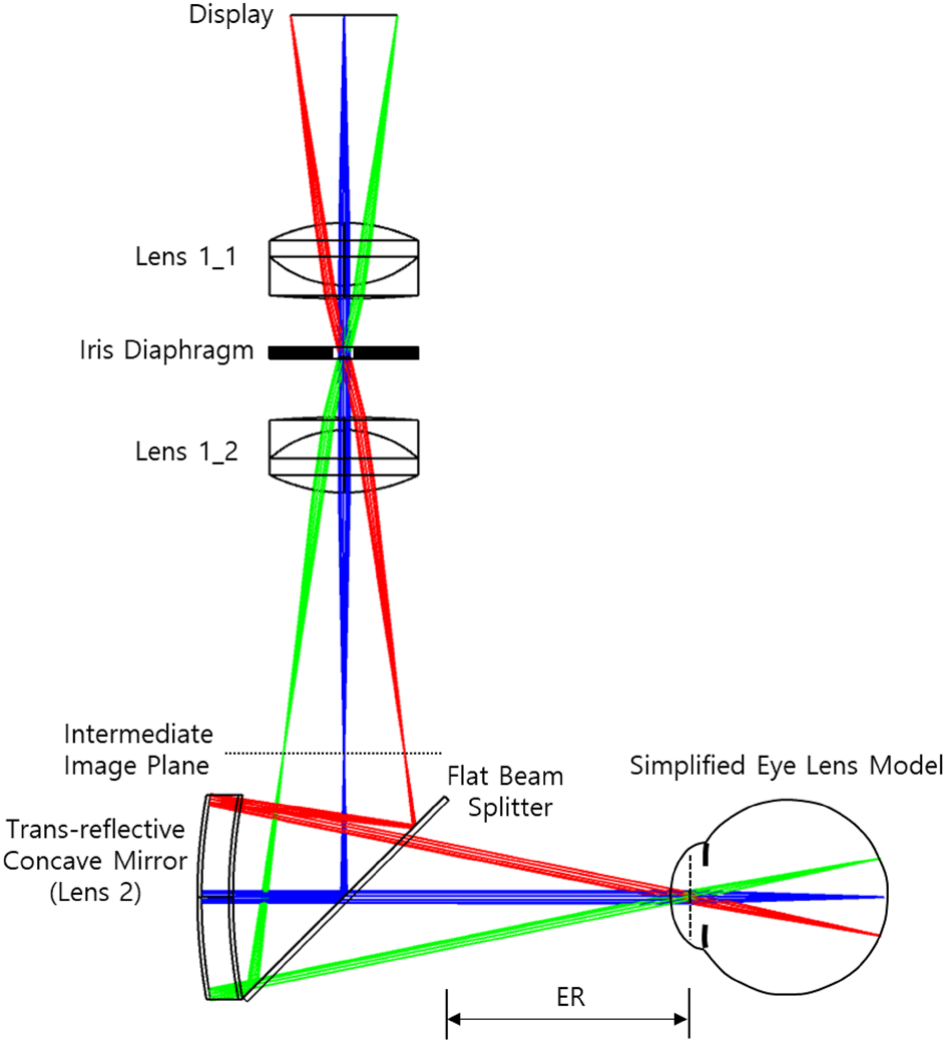
AR optics combined with an expanded DOF module.

For verification, an AR optical system with a DOF range of 3.0 D (diopter) and an HFOV of 35.3 degrees is designed. In addition, in a quantitative method, we analyze the quality of the virtual images in the designed AR optical system with a simplified eye lens model. The configuration of the AR optical system for the verification of the DOF range is shown in Fig. 5. Specific specifications of Fig. 5 are given in the Table 1.

**Table 1:** Specifications of optical elements and AR optics.

Items	Specification
Display	FHD (1920 × 1080) OLED
Lens 1_1 and 1_2	Achromatic, focal length 20 mm
2nd lens (trans-reflective concave mirror)	Focal length 24.8 mm
Used image resolution	1458 × 820 pixels
HFOV	35.3 degree
Eye relief (ER)	19.1 mm
Best focus position	1.5 Diopter (0.67 m)
PD/DOF range	0.89 mm, 1.08 mm, 1.53 mm, 3.0 mm/3.0 Diopter, 2.0 Diopter, 1.0 Diopter, 0.26 Diopter
Corresponding diffraction limit optical system	
Do	64.84 mm
De	40.16 mm
Do:De	1.61:1
1st lens	EFFL 17.46 mm
Main optics lens	EFFL 24.8 mm
Simplified eye lens model	Single paraxial lens (EFFL 16 mm) and variable image plane position for focus adjustment

**Figure 6:**
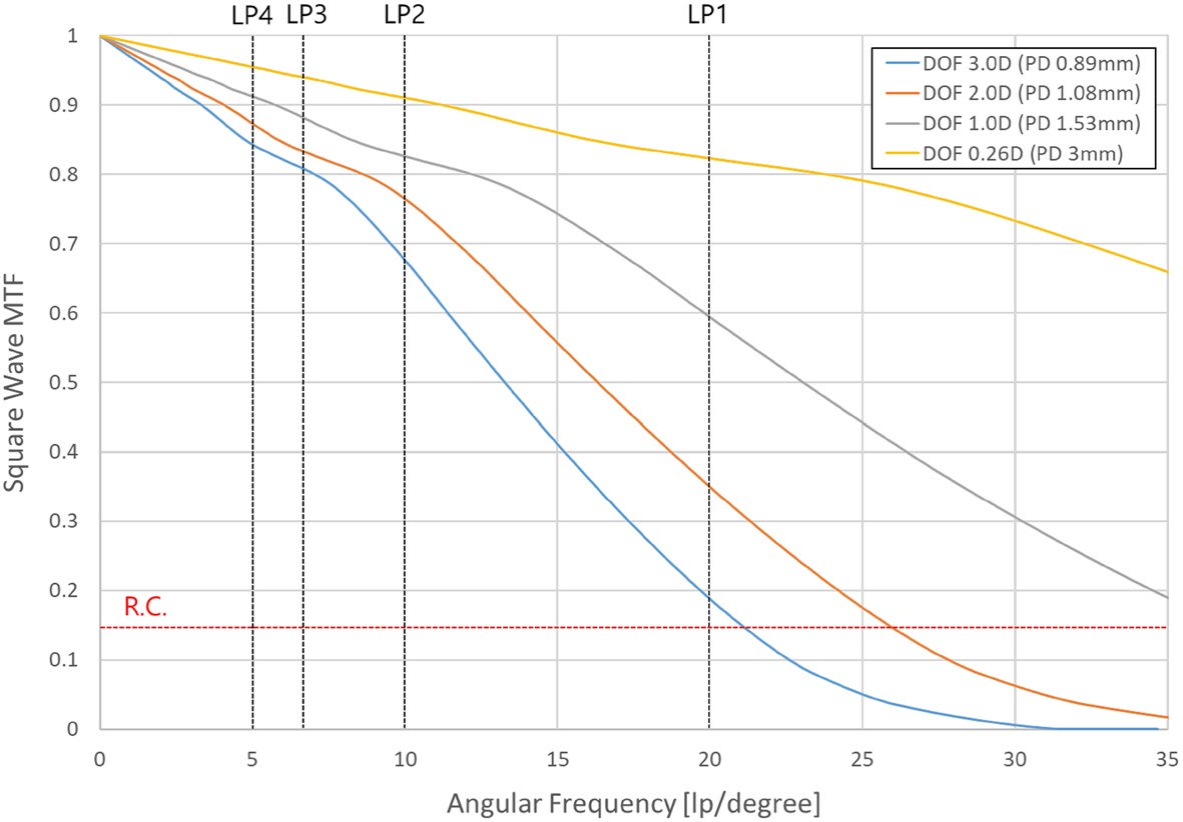
Square wave MTF characteristics in 4 types of PD conditions.

The simulation result of whether the DOF range can be effectively controlled by adjusting the PD in the previously designed optical system is shown in Fig. 6, where three color wavelengths (0.4861 µm, 0.5876 µm, 0.6563 µm) are used for comparison with the experiment and the MTF characteristics of zero-field for square waves are examined since a virtual image is from a display with pixels.

**Figure 7:**
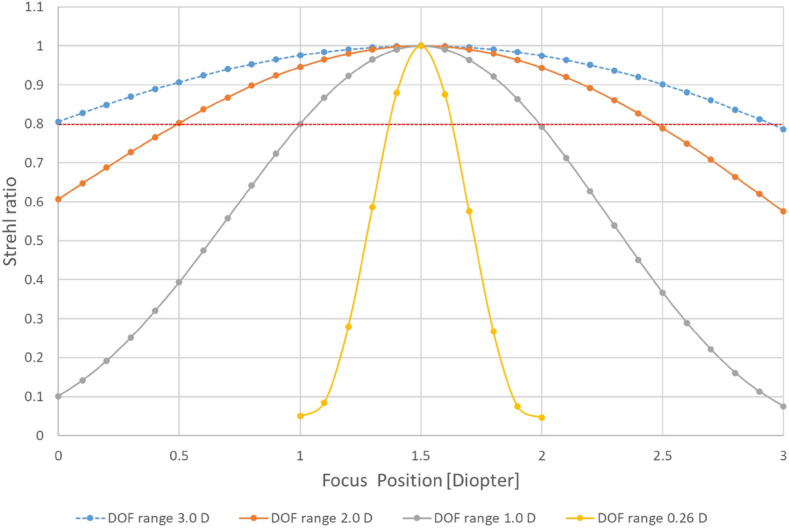
Strehl ratio characteristics of the PSF according to eye focus control for each PD condition.

The angular frequencies corresponding to line pairs (LPs) of the virtual image are represented by vertical dotted lines, and the MTF value (~ 0.14) calculated from the maximum and minimum values of modulation in the Rayleigh criterion condition for the adjacent PSF is indicated by the horizontal dotted red line^53^. The values of PDs in the simulation are set to be the PD size in which the DOF range calculated by Eq. (5) are 3D, 2D, 1.0D, and 0.26D, respectively. LP1 is configured with on/off of a unit pixel, LP2 is configured with on/off of two adjacent unit pixels, LP3 is configured with on/off of three adjacent unit pixels, and LP4 is configured with on/off of four adjacent unit pixels. As shown in Fig. 6, the resolution decreases when the PD decreases to increase the DOF range. For the PD of 0.89 mm corresponding to the DOF range of 3D, the MTF contrast at the angular frequency of 20 cpd for the LP1 pattern is approximately 0.19, which is a sufficiently larger contrast value than the condition of the Rayleigh criterion. When the focus adjustment position of the simplified eye model for each PD condition is changed from the best focus (1.5D) by a 0.1D step, the results of simulating the change in the Strehl ratio of the PSF are shown in Fig. 7.

**Figure 8:**
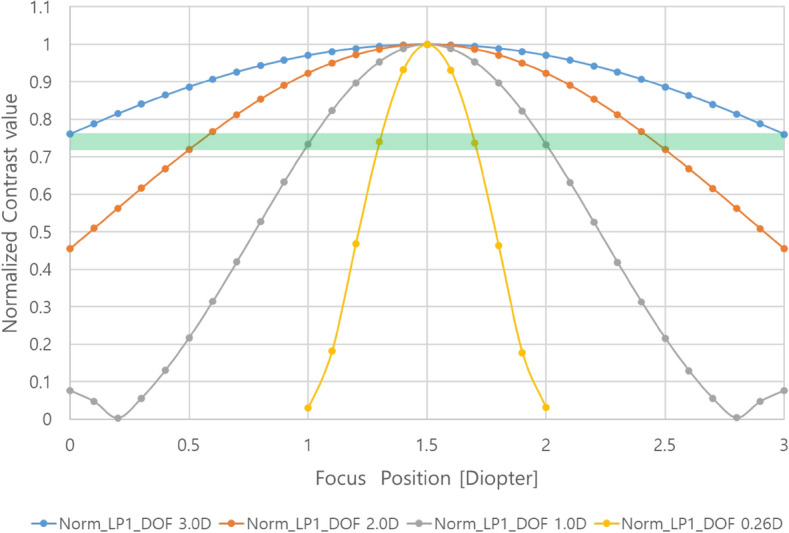
Normalized square wave MTF characteristics of LP1 patterns.

As mentioned before, the case in which the Strehl ratio of the PSF is greater than 0.8 corresponds to the in-focus region. It is shown in Fig. 7 that the range in which the Strehl ratio is 0.8 or higher for each PD condition set by calculation is almost the same as the DOF range determined by the calculation. To confirm the relevant characteristics indirectly, the simulation result for the contrast value of the LP1 pattern corresponding to the highest resolution of the virtual image is shown in Fig. 8, where the contrast value is normalized to 1 to compare the rate of change of the contrast value for each PD condition. Compared with the Strehl ratio for each focusing position of the eye, it can be checked that the normalized contrast value of 0.71 to 0.77 corresponds to the Strehl ratio of 0.8, although it is not the same contrast value for each condition. The reason why these have not the exact same value but a certain range is that the resolution is different for each PD condition and the sensitivity of the contrast value is different even at the same value of the Strehl ratio according to focus adjustment.

**Figure 9:**
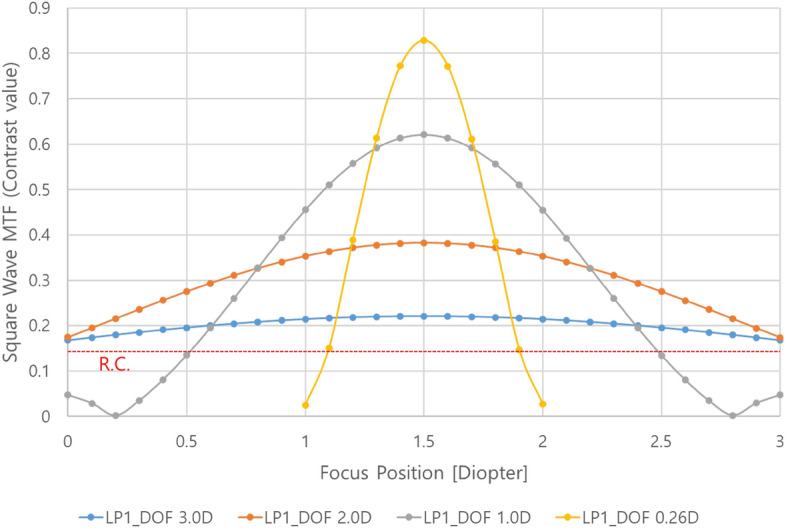
Square wave MTF characteristics of LP1 patterns.

The characteristics for the absolute value of the contrast of the LP1 pattern, which is the highest resolution of the virtual image, according to the focus adjustment are shown in Fig. 9. It is also shown in Fig. 9 that the focus control range of the LP1 pattern recognized from the Rayleigh criterion (contrast value ~ 0.14) is designed to be wider than the DOF range based on the criterion of the Strehl ratio defined before.

### Experimental verifications

**Figure 10:**
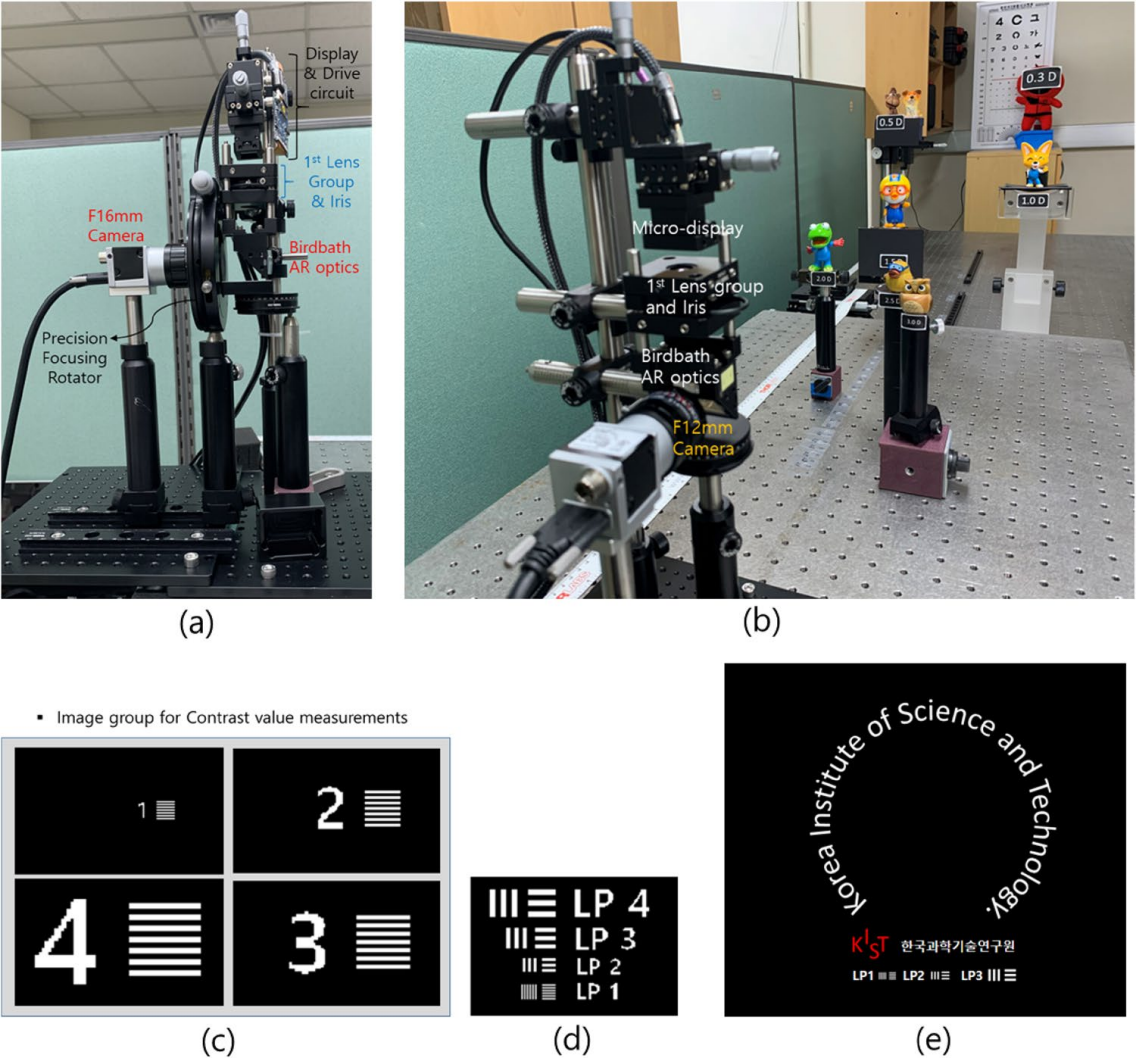
EDOF AR optic system setting pictures and images for the experiment.

The designed AR optical system is constructed, and the experimental results are quantitatively and qualitatively analyzed by using a fixed focal length camera that replaces the virtual image recognition of the eye. As shown in Fig. 10, the AR optical system with the expanded DOF (EDOF) module for the experimental verification is configured according to the specifications in Table 1.

Figure 10a is a measurement system for quantitative evaluation. For the MTF measurement experiment, the images in Fig. 10c, in which the LP1, LP2, LP3, and LP4 patterns are arranged on the optical axis, are used to obtain the quantitative MTF measurement value. In addition, the images for the qualitative comparison evaluation of the MTF pattern according to the conditions of the DOF range are used in the images of Fig. 10d,e, in which LP1–LP4 patterns are arranged.

**Figure 11:**
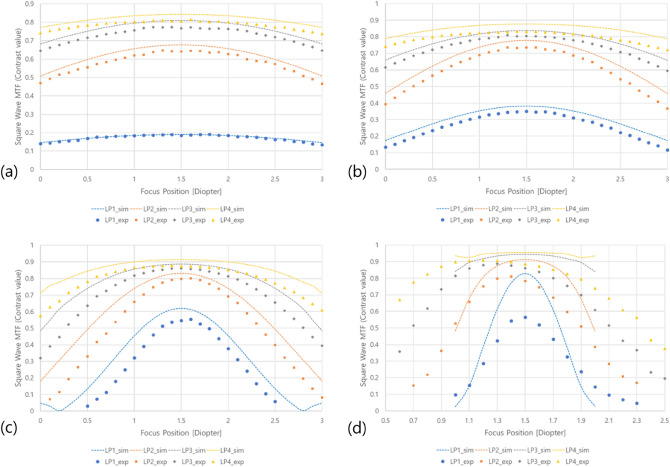
Comparison of contrast value characteristics simulation and experimental results according to focus control for each aperture condition. (**a**) PD 0.885 mm, (**b**) PD 1.084 mm, (**c**) PD 1.533 mm, (**d**) PD 3 mm.

To determine whether the DOF range determined by the calculation for each aperture condition is experimentally validated, the contrast value is experimentally measured from the images taken by changing the focus control of the camera in units of 0.1D for the four spatial frequencies of the virtual image. The simulation results of the designed optical system are compared in Fig. 11.

The simulation and experimental results of the contrast values according to the camera focusing position for each of the four spatial frequencies according to PD reasonably match each other under the PD condition within 2 mm. Except for the condition that PD is 3 mm, it is shown that the contrast at the best focus is maintained in a wide focus range when PD decreases, as predicted by simulation.

**Figure 12:**
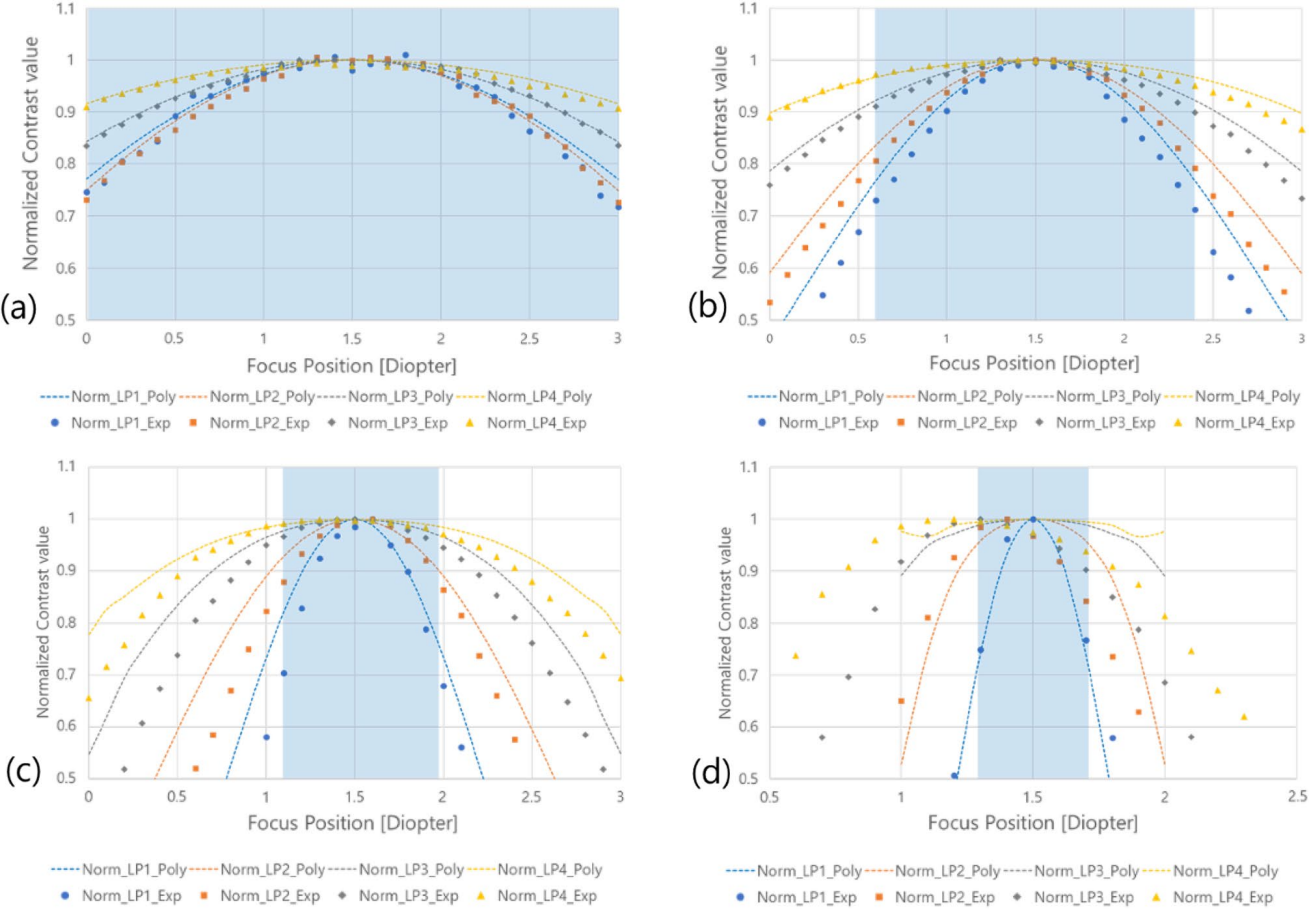
Comparison of normalized contrast value characteristics simulation and experimental results according to focus control for each aperture condition. (**a**) PD 0.885 mm, (**b**) PD 1.084 mm, (**c**) PD 1.533 mm, (**d**) PD 3 mm.

Figure 12 represents the comparison of results obtained by normalizing the change in the contrast value according to the focus adjustment in the simulation and the experiment of Fig. 11. In this experiment, the value of the Strehl ratio as logical evidence to determine DOF cannot be directly measured. However, it is experimentally shown that the DOF range according to the PD size can be adjusted from a range where the normalized contrast value corresponding to the value is changed.

**Figure 13:**
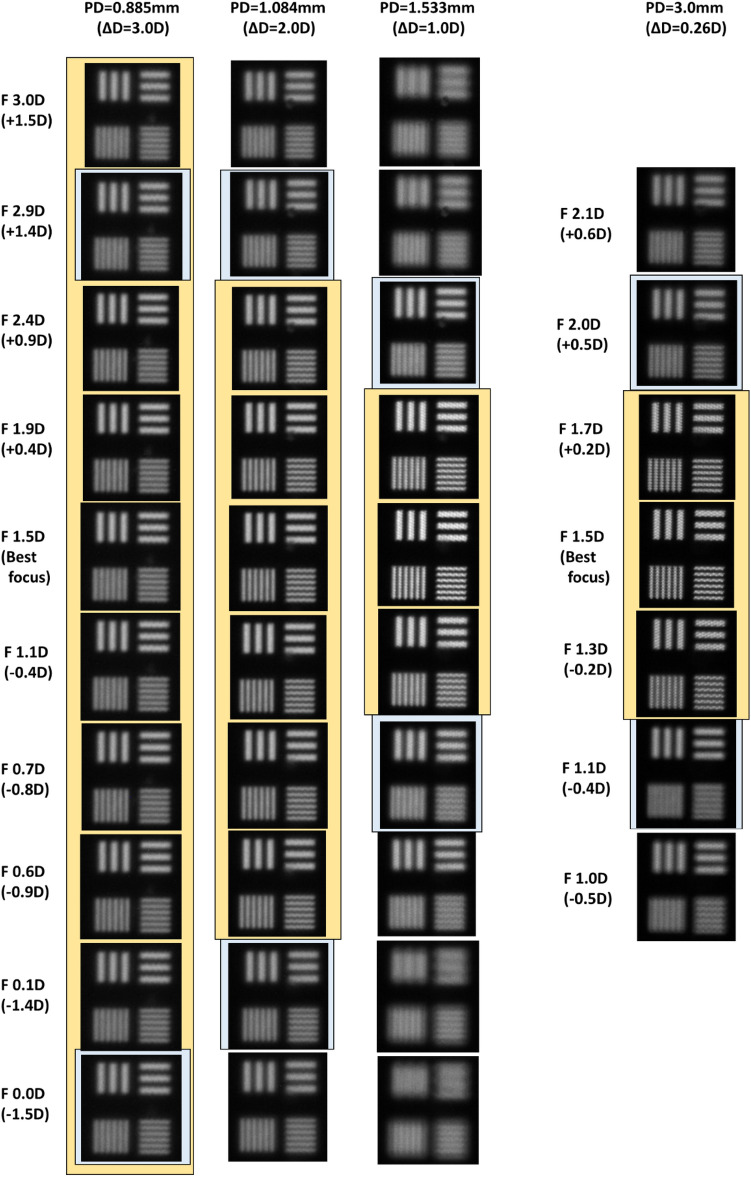
MTF pattern results for each PD condition according to focus adjustment.

The MTF pattern of LP1 and LP2 among the results captured the image in Fig. 10d, adjusting the focal depth of camera in the designed AR optical system for verification, is represented in Fig. 13, where the qualitative results of captured images for DOF range are shown according to PD size. In the cases of a PD of 0.885 mm for the DOF condition of 3.0D, a PD of 1.084 mm for the DOF condition of 2.0D, a PD of 1.533 mm for the DOF condition of 1.0D, and a PD of 3 mm for the DOF condition of 0.26D, the MTF pattern images are compared by adjusting the focal depth of the camera. In the image for each focal depth condition in Fig. 13, the images in the yellow background indicate the images within the DOF range based on the standard Strehl ratio, and the images in the blue background indicate that the images meet the Rayleigh criterion condition. When the PD is 0.885 mm, even if the focus changes within ± 1.5 D of the best focus (1.5D), which is the DOF range based on the standard Strehl ratio, there is no significant degradation of image quality compared to the optimal image, so it is expected that the VAC caused by the inconsistency between convergence and the focus adjustment of the eyes can be alleviated. In the case of a PD of 1.084 mm, it is shown that the degradation of image quality compared to the optimal image is not significantly felt in the ± 0.9D range of the best focus (1.5D), which is 0.2D smaller than the DOF range based on the standard of the Strehl ratio. When the PD is 1.53 mm, the deterioration of image quality is not felt much compared to the optimal image in the ± 0.4D range from the best focus (1.5D), which is the DOF range based on the standard Strehl ratio. The LP1 pattern in the focus adjustment area between + 0.9D and − 0.8D from the best focus (1.5D) provides a higher contrast than the Rayleigh criterion. The conditions of a conventional AR optical system are similar to the condition that the PD is more than 3 mm. The degradation of image quality is hardly felt at approximately ± 0.2D position of best focus compared to image quality at the optimal focus position, but the image quality deteriorates rapidly above that.

**Figure 14:**
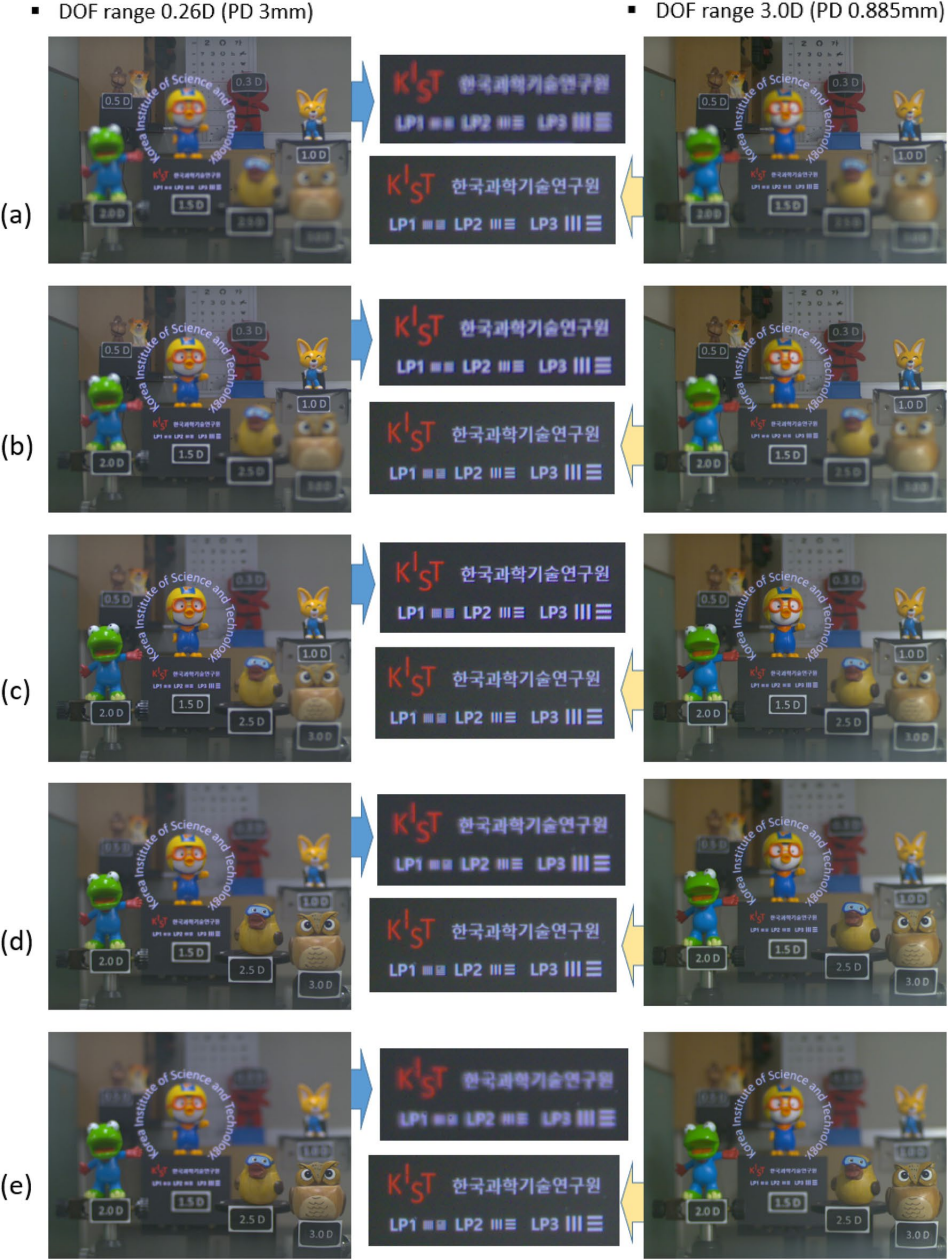
Comparison of virtual image and real object according to focus adjustment at PD 3 mm and PD 0.885 mm. Focus on (**a**) 0.0D, (**b**) 0.5D, (**c**) 1.5D, (**d**) 2.5D, and (**e**) 3.0D.

The optical system for performing the qualitative analysis is shown in Fig. 10b. In the experimental arrangement from 3.0D to 0.3D, real objects are placed, and 0D (Infinite distance) is set as the estimated focal depth using the lens focusing rotation angle of the camera. In addition, the image of Fig. 10e is used for the virtual image. The letters arranged in a circle are set to the size corresponding to the LP4 pattern, and the first sentence arranged in the horizontal direction is set to the size corresponding to the LP3 pattern. The LP1, LP2, and LP3 patterns are placed in the second row for comparison with the quantitative analysis. A comparison between captured images of the virtual image and real objects according to focal depth adjustment is shown in Fig. 14 for the condition that PDs are 3 mm and 0.885 mm. In Fig. 14, the images for the PD condition of 3 mm corresponding to the conventional AR glasses condition are on the left, and the images for the PD condition of 0.885 mm corresponding to the DOF range of 3.0D are on the right. In the conventional PD condition of 3 mm, as analyzed in the quantitative evaluation, it can be checked that the virtual image of the horizontal text deteriorates from the focal depth ± 1.0D away from the optimal focal depth. This result implies the low DOF phenomenon that can appear in the case of the conventional AR optical system. In this paper, as a qualitative result of the evidence that the DOF can be expanded by adjusting the PD size at the eye pupil position, the typical result for the PD size corresponding to the DOF range of 3.0D is shown on the right side of Fig. 14. Unlike the result on the left side in Fig. 14, even if the focus is adjusted to ± 1.5D from 1.5D, it can be found that the LP1 pattern of 20 cpd is resolvable. To clearly show the difference in picture quality of the virtual image in the setting condition of when DOF is 3D and the conventional conditions according to such focus adjustment, the photos in the middle row of Fig. 14 are enlarged photos of the text part of the virtual image.

(Qualitative DOF ranges for the left and right parts of Fig. 14 can be found as Supplementary Videos S1 and S2).

## Discussion

In relation to monocular focusing, which is one of the important factors in implementing a 3D display, the conditions for the expansion of DOF are derived. If these fundamental conditions are implemented in the full parallax-type SMV method, focus adjustment can be simulated artificially so that an image similar to a hologram can be realized. It is also possible to develop 3D displays that are all-in-focus by applying only a single parallax image having a wide DOF condition to a single eye. Practically, a wide DOF can be more easily realized in a narrow viewing area, such as a near-eye display. For this reason, it has been first applied to the AR optical system to implement a wide DOF of 3.0 diopter range. It has been confirmed that the DOF range can be used as an appropriate standard by introducing the Rayleigh criterion and the standard of the Strehl ratio. By combining the AR optical system with the extended DOF module that provides the wide DOF in this paper, it has been shown that the FOV is determined in consideration of the diffraction effect and the geometric defocus effect, and the DOF range and resolution can be adjusted according to the PD and the size of the convergence area in the eye pupil plane. It has also been shown that the effect of improving resolving power can be achieved by adjusting the PD according to the situation where the virtual image is provided. If these characteristics are properly used, it is possible to provide a wide DOF and high-resolution virtual images in various XR applications. An optical standard could be presented to provide comfortable 3D images without VAC. Therefore, it is expected that these results can also be utilized in the optical design and implementation of glasses-free 3D displays in the future. Additionally, in future studies, the DOF limitation due to diffraction, which hinders the wide application of DOF expansion, and how to enlarge the eye box area^36,38,56,57^ will be important topics.

## Materials and methods

### AR optics construction and verification

In Fig. 5, two FL 20 mm achromatic lenses are used to minimize the geometrical aberration and color aberration. In addition, the size of the PD is controlled by adjusting the iris diaphragm located in the middle of the two achromatic lenses. This optical system combined with the display is the EDOF module. The image of the display passes through the EDOF module, and an intermediate image is generated before the AR optics. Then, this intermediate image passes through the AR optical system to form a converging region on the pupil plane of the eye and finally forms an image in the retina. The AR optical system is a birdbath-type AR optical system consisting of a transreflective concave mirror and a flat beam splitter. The HFOV and eye relief in the designed AR optical system are 35.3 degrees and 19.1 mm, respectively, as shown in Fig. 5 and Table 1. For this, the virtual image that can be seen from the eye position is 57.7% of the FHD display area. The optimal depth of the virtual image plane of the optical system is designed to be 1.5 Diopter. The size of the iris diaphragm is adjusted to alter the PD size on the pupil plane of the eye. The DOF range is shown in Table. 1 can be adjusted according to the PD size. The simplified eye lens model used in the simulation consists of a single paraxial lens with a focal length of 16 mm, close to 60 dioptres, which is the equivalent power of the eye lens. It is configured to adjust the position of the image plane corresponding to the focal depth adjustment^58^.

Figure 10a is a measurement system for quantitative evaluation where the camera (Model acA2500-14 µm) with an F 16 mm lens (No. 59870) of Edmund Optics and a 1/2.5″ CMOS sensor of Basler is used. The camera sensor is selected to have a pixel size of 2.2 µm to reflect the characteristics of the eye. The aperture conditions are set to be values of PD on the pupil of the eye corresponding to the DOF extensions of 3.0D, 2.0D, 1.0D, and 0.26D. In the experiment for these conditions, the optimal focal depth is set to 1.5D. For the DOF extension experiment, changing the focus adjustment position of the camera in units of 0.1D, the MTF pattern images are captured by the camera. In addition, for the MTF measurement experiment, the images in Fig. 10c, in which the LP1, LP2, LP3, and LP4 patterns are arranged on the optical axis, are used to obtain the quantitative MTF measurement value, and the contrast values are calculated from the maximum and minimum values for each pattern. In addition, the images for the qualitative comparison evaluation of the MTF pattern according to the conditions of the DOF range are used in the images of Fig. 10d,e, in which LP1–LP4 patterns are arranged.

## Supplementary Information

Supplementary Legends.

Supplementary Video 1.

Supplementary Video 2.

## Data Availability

The datasets used and/or analysed during the current study available from the corresponding author on reasonable request.

## References

[1] Okoshi T (1976). Three Dimensional Imaging Techniques.

[2] Lueder E (2012). 3D Displays.

[3] Sandin DJ, Margolis T, Ge J, Girado J, Peterka T, DeFanti TA (2005). The Varrier TM autostereoscopic virtual reality display. ACM Trans. Graph..

[4] Kim S-K, Yoon K-H, Yoon SK, Ju H (2015). Parallax barrier engineering for image quality improvement in an autostereoscopic 3D display. Opt. Express.

[5] Kim SK, Yoon KH, Yoon SK, Ju H (2015). Defragmented image based autostereoscopic 3D displays with dynamic eye tracking. Opt. Commun..

[6] Kang M-K, Nguyen H-P, Kang D, Park S-G, Kim S-K (2018). Adaptive viewing distance in super multi-view displays using aperiodic 3-D pixel location and dynamic view indices. Opt. Express.

[7] Dodgson NA (2005). Autostereoscopic 3D displays. IEEE Comput. Soc..

[8] Yoshihiro K, Hiroshi Y, Toshio H (1997). Hologramlike video images by 45-view stereoscopic display. Proc. SPIE.

[9] Takaki Y, Nago N (2010). Multi-projection of lenticular displays to construct a 256-view super multi-view display. Opt. Express.

[10] Hoshino H, Okano F, Isono H, Yuyama I (1998). Analysis of resolution limitation of integral photography. J. Opt. Soc. Am. A.

[11] Son J-Y, Son W-H, Kim S-K, Lee K-H, Javidi B (2012). Three-dimensional imaging for creatin real-world-like environments. Proc. IEEE.

[12] Campbell FW (1957). The depth of field of the human eye. J. Mod. Opt..

[13] Lambooij M, Jsselsteijn W (2009). Visual discomfort and visual fatigue of stereoscopic displays: A review. J. Imaging Sci. Technol..

[14] Hoffman DM, Girshick AR, Akeley K, Banks MS (2008). Vergence-accommodation conflicts hinder visual performance and cause visual fatigue. J. Vis..

[15] Huebschman ML, Munjuluri B, Garner HR (2003). Dynamic holographic 3-D image projection. Opt. Express.

[16] An J, Won K, Kim Y, Hong J-Y, Kim H, Kim Y, Song H, Choi C, Kim Y, Seo J, Morozov A, Park H, Hong S, Hwang S, Kim K, Lee H-S (2020). Slim-panel holographic video display. Nat. Commun..

[17] Blundell B, Schwartz A (1999). Volumetric Three-Dimensional Display Systems.

[18] Downing E, Hesselink L, Ralston J, Macfarlanem R (1996). A three-color, solid-state, three-dimensional display. Sci. Res..

[19] Miyazaki D, Shiba K, Sotsuka K, Matsushita K (2006). Volumetric display system based on three dimensional scanning of inclined optical image. Opt. Express.

[20] Kramida G (2016). Resolving the vergence-accommodation conflict in head-mounted displays. IEEE TVCG.

[21] Matsuda N, Fix A, Lanman D (2017). Focal surface displays. ACM Trans. Graph..

[22] Hua H (2017). Enabling focus cues in head-mounted displays. Proc. IEEE.

[23] Maimone A, Lanman D, Rathinavel K, Keller K, Luebke D, Fuchs H (2014). Pinlight displays: Wide field of view augmented reality eyeglasses using defocused point light sources. ACM Trans. Graph..

[24] Ueno T, Takaki Y (2018). Super multi-view near-eye display to solve vergence-accommodation conflict. Opt. Express.

[25] Huang F-C (2015). The light field stereoscope immersive computer graphics via factored near-eye light field displays with focus cues. ACM Trans. Graph..

[26] Huang H, Hua H (2019). Effects of ray position sampling on the visual responses of 3D light field displays. Opt. Express.

[27] Liu S, Hua H, Cheng D (2010). A novel prototype for an optical see-through head-mounted display with addressable focus cues. IEEE Trans. Vis. Comput. Graph..

[28] Lee S, Jo Y, Yoo D, Cho J, Lee D, Lee B (2019). Tomographic near-eye displays. Nat. Commun..

[29] Kim S-K, Kim D-W, Kwon YM, Son J-Y (2008). Evaluation of the monocular depth cue in 3D displays. Opt. Express.

[30] Kim S-B, Park J-H (2018). Optical see-through Maxwellian near-to-eye display with an enlarged eyebox. Opt. Lett..

[31] Do H, Kim YM, Min S-W (2019). Focus-free head-mounted display based on Maxwellian view using retroreflector film. Appl. Opt..

[32] Jang C, Bang K, Moon S, Kim J, Lee S, Lee B (2017). Retinal 3D: Augmented reality near-eye display via pupil-tracked light field projection on retina. ACM Trans. Graph..

[33] Westheimer G (1966). The Maxwellian view. Vis. Res..

[34] Kim D-W, Kwon Y-M, Park Q-H, Kim S-K (2011). Analysis of a head-mounted display-type multifocus display system using a laser scanning method. Opt. Eng..

[35] Kim S-K, Kim E-H, Kim D-W (2011). Full parallax multifocus three-dimensional display using a slanted light source array. Opt. Eng..

[36] Zhang Q, Piao Y, Ma S, Liu Y, Wang Y, Song W (2022). Design, analysis and optimization of a waveguide-type near-eye display using a pin-mirror array and a concaved reflector. Opt. Express.

[37] Park S (2021). Augmented and mixed reality optical see-through combiners based on plastic optics. Inf. Display.

[38] Choi M-H, Shin K-S, Jang J, Han W, Park J-H (2022). Waveguide-type Maxwellian near-eye display using a pin-mirror holographic optical element array. Opt. Lett..

[39] Wang Z, Tu K, Pang Y, Xu M, Lv G, Feng Q, Wang A, Ming H (2022). Lensless phase-only holographic retinal projection display based on the error diffusion algorithm. Opt. Express.

[40] Song W, Li X, Zheng Y, Liu Y, Wang Y (2021). Full-color retinal-projection near-eye display using a multiplexing-encoding holographic method. Opt. Express.

[41] Takaki Y, Fujimoto N (2018). Flexible retinal image formation by holographic Maxwellian-view display. Opt. Express.

[42] Lee JS, Kim YK, Lee MY, Won YH (2019). Enhanced see-through near-eye display using time-division multiplexing of a Maxwellian-view and holographic display. Opt. Express.

[43] Westheimer G (1959). Retinal light distribution for circular apertures in Maxwellian view. J. Opt. Soc. Am..

[44] Jacobs RJ, Bailey IL, Bullimore MA (1992). Artificial pupils and Maxwellian view. Appl. Opt..

[45] Zalevsky Z (2010). Extended depth of focus imaging: A review. SPIE Rev. Soc. Photo-Opt. Instrum. Eng..

[46] Zhao J, Xia J, Ma Q, Wu J (2019). Spatial loss factor for the analysis of accommodation depth cue on near-eye light field displays. Opt. Express.

[47] Havarn SNV, Hosek J (2023). Different view on diffraction-limited imaging optics design. J. Opt. Soc. Am. A.

[48] Miller J (2006). Depth-of-focus of the human eye: Theory and clinical implications. Surv. Ophthalmol..

[49] Goodman JW (1996). Introduction to Fourier Optics.

[50] Charman WN, Charman WN (1995). Optics of the eye. Handbook of Optics.

[51] Geary JM (2002). Introduction to Lens Design.

[52] Smith WJ (1991). Modern Optical Engineering.

[53] Fischer R, Tadic B (2000). Optical System Design.

[54] Sinzinger S, Jahns J (2006). Microoptics.

[55] Török P, Kao F-J (2013). Optical Imaging and Microscopy: Techniques and Advanced Systems.

[56] Kim, S. K. & Park, S. *Full Parallax Multi-focus Three-Dimensional Display. US Patent, No. US 10,666,930 B2* (2020).

[57] Kim, S. K. *Near-Eye Display Device. US Patent, Pub. No. US 2023/0048195 A1* (2023).

[58] Smith G, Atchison DA (1997). The Eye and Visual Optical Instruments.

