# Supplementary material for: Extended depth of field in augmented reality
Source: Sci Rep. 2023 May 31;13:8786. doi: 10.1038/s41598-023-35819-9 (PMC10232407; doi:10.1038/s41598-023-35819-9)
Supplement: Supplementary file 1 — Supplementary Legends. [file 41598_2023_35819_MOESM1_ESM.docx]

Supplementary video1 : Supplementary Video S1.mp4 (Qualitative DOF range of PD 3mm)

This video includes qualitative testing of the DOF range (~1.5D ± 0.5D) under PD 3mm condition.

Real small animal models are at different depths (3.0D~0.3D). And, on the virtual screen with a depth of 1.5D, the circular text (Korea Institute of Science and Technology) and the patterns of LP1, LP2, and LP3 are virtual images for qualitative resolution test.

Supplementary video2 : Supplementary Video S2.mp4 (Qualitative DOF range of PD 0.885mm)

This video includes qualitative testing of the DOF range (~1.5D ± 1.5D) under PD 0.885mm condition.

Real small animal models are at different depths (3.0D~0.3D). And, on the virtual screen with a depth of 1.5D, the circular text (Korea Institute of Science and Technology) and the patterns of LP1, LP2, and LP3 are virtual images for qualitative resolution test.
